# Transcriptomic and metabolic responses of *Staphylococcus aureus *exposed to supra-physiological temperatures

**DOI:** 10.1186/1471-2180-9-76

**Published:** 2009-04-22

**Authors:** Bénédicte Fleury, William L Kelley, Daniel Lew, Friedrich Götz, Richard A Proctor, Pierre Vaudaux

**Affiliations:** 1 Service of Infectious Diseases, Geneva University Hospital, 4 rue Gabrielle-Perret-Gentil, CH-1211 Geneva 14, Switzerland; 2 Microbial Genetics, University of Tübingen, Tübingen, Germany; 3 Department of Medical Microbiology/Immunology, University of Wisconsin School of Medicine and Public Health, Madison, WI, USA; 4 Current address: Merck & Co Inc, 351 North Sumneytown Pike, MS-UG4C-48 – UG3D-001, North Wales, PA 19454, USA

## Abstract

**Background:**

Previous evaluation by different molecular and physiological assays of *Staphylococcus aureus *(*S. aureus*) responses to heat shock exposure yielded a still fragmentary view of the mechanisms determining bacterial survival or death at supra-physiological temperatures. This study analyzed diverse facets of *S. aureus *heat-shock adjustment by recording global transcriptomic and metabolic responses of bacterial cultures shifted for 10 min from 37°C to a sub-lethal (43°C) or eventually lethal (48°C) temperature. A relevant metabolic model of the combined action of specific stress response mechanisms with more general, energy-regulating metabolic pathways in heat-shocked *S. aureus *is presented.

**Results:**

While *S. aureus *cultures shifted to 43°C or left at 37°C showed marginal differences in growth and survival rates, bacterial cultures exposed to 48°C showed a rapid growth arrest followed by a subsequent decline in viable counts. The most substantial heat shock-induced changes at both 43°C and 48°C occurred in transcript levels of HrcA- and CtsR-regulated genes, encoding classical chaperones DnaK and GroESL, and some Hsp100/Clp ATPases components, respectively. Other metabolic pathways up-regulated by *S. aureus *exposure at 48°C included genes encoding several enzymes coping with oxidative stress, and DNA damage, or/and impaired osmotic balance. Some major components of the pentose phosphate cycle and gluconeogenesis were also up-regulated, which reflected depletion of free glucose by bacterial cultures grown in Mueller-Hinton broth prior to heat shock. In contrast, most purine- and pyrimidine-synthesis pathway components and amino acyl-tRNA synthetases were down-regulated at 48°C, as well as arginine deiminase and major fermentative pathway components, such as alcohol, lactate and formate dehydrogenases. Despite the heat-induced, increased requirements for ATP-dependent macromolecular repair mechanisms combined with declining energy sources, intracellular ATP levels remained remarkably constant during heat shock.

**Conclusion:**

The sequential loss of replication and viability at 48°C cannot be explained by significant reductions in intracellular ATP levels, but may reflect ATP rerouting for macromolecular repair mechanisms and cell survival. Our metabolic model also suggests that heat-stressed *S. aureus *should down-regulate the production of potential, DNA-damaging reactive oxygen species that might result from electron transport-generated ATP, involving excessive levels of free heavy metals, in particular iron.

## Background

*Staphylococcus aureus *is a versatile pathogen that can cause a wide spectrum of localized or disseminated diseases [1,2], as well as colonizing healthy carriers [3,4]. The mechanisms that may explain *S. aureus *physiological and pathogenic versatility are: (i) acquisition and exchange of a number of mobile genetic elements (carrying different toxins, antibiotic resistance determinants, others) by horizontal intra- or interspecies transfer [5]; (ii) the presence of highly elaborated signal-transduction and regulatory pathways, including at least one quorum-sensing system [6], which are coordinated by a number of global regulators that respond to environmental or host stimuli [6-9]; and (iii) the contribution of elaborated stress response systems to severe environmental conditions such as oxidant injury, extremes in pH and temperature, metal ion restriction, and osmotic stress [10].

Molecular chaperones or proteases involved in the refolding or degradation of stressed, damaged proteins, many of which are classed as heat shock proteins (HSP), play important roles in bacterial stress tolerance [11,12]. Comparative genomic studies with *B. subtilis *allowed the identification two major, chaperone-involving stress response pathways in *S. aureus *[8,13]. The first category includes genes encoding classical chaperones (DnaK, GroES, GroEL) that modulate protein folding pathways, in either preventing misfolding and aggregation or promoting refolding and proper assembly [12]. While these classical chaperones, such as DnaK and GroESL, are widely conserved among gram-negative and gram-positive bacterial species, their detailed physiological function was little studied in *S. aureus *until recently [14]. The second category includes *clpC*, *clpB*, and *clpP *coding for combined chaperone and ATP-dependent protease activities [13], also referred to as the family of Hsp100/Clp ATPases and proteases, whose activity was mostly studied in *B. subtilis *and *E. coli *[12]. By homology, the proteolytic activity in *S. aureus *is assumed to occur inside hollow, barrel-shaped "degradation chambers", composed of ClpP protease oligomers associated with Hsp100/Clp ATPases, non-proteolytic chaperone components that specifically recognize proteins tagged for disassembly, unfolding, and/or degradation [12]. The major global regulatory impact of the ClpP protease family on *S. aureus *physiology and metabolism was recently evaluated by a combined approach of genetic knockout and transcription profiling [15]. Several other studies demonstrated the pleiotropic phenotypic effects of individual knockout mutations in *S. aureus clpX*, *clpC*, *clpB*, *clpL*, ATP-dependent chaperones, which affected virulence in animal models, biofilm formation, endocytosis, cell wall autolysis, and resistance to stress exposure [16-18]. These genetic studies demonstrated the complex molecular interactions of stress response mechanisms, occurring at both transcriptional and post-translational levels [15-18]. While *clpC*, *clpB*, and *clpP *are controlled by the CtsR repressor, the HrCA regulon (*dnaK *and *groESL *operons) of *S. aureus *was found embedded within the CtsR regulon, in contrast to *B. subtilis*, which might provide a tighter control of major heat shock regulons in *S. aureus *[13,19].

Initially considered as a major stress response system that would help to face diverse stressful stimuli (including some antibiotics) [20,21], the SigB regulon is now believed to have a more general physiological impact on *S. aureus *compared to *B. subtilis *or *E. coli*, influencing ca. 200 genes involved in several cellular processes such as cell envelope composition, membrane transport processes, and intermediary metabolism [22,23]. The SigB operon of *S. aureus *is composed of four ORFs (*rsbU*, *rsbV*, *rsbW*, *sigB*), coding for the regulatory network components of transcriptional factor sigma B activity (SigB) [20,21,24,25]. Evaluation of intracellular levels and functional activity of free SigB is achieved by assaying transcription of the SigB-dependent target gene *asp23 *[26]. Previous studies have shown that *S. aureus *strain NCTC8325 and its in vitro-generated derivatives are defective in RsbU expression thus impairing post-transcriptional, upregulation of free SigB by external or internal stimuli [27-29].

In the past decades, *S. aureus *responses to heat shock exposure were evaluated by a variety of molecular and physiological assays, which yielded a still fragmentary view of the mechanisms determining bacterial survival or death at supra-physiological temperatures [14,30-33]. This report aims to analyze diverse facets of *S. aureus *stress responses to heat exposure, by evaluating in parallel the combined action of specific stress response mechanisms with more general, energy-regulating metabolic pathways. The short term physiological adjustment of *S. aureus *from 37°C to higher temperatures was evaluated by recording the global transcriptomic responses of bacterial cultures briefly exposed (10 min) to one sub-lethal (43°C) and one eventually lethal (48°C) temperature, in parallel with determination of some major intracellular and extracellular markers of metabolic pathways regulating energy sources and microbial cell viability.

## Results and discussion

### Global analysis of transcriptomic responses

To evaluate the impact of temperature up-shifts on the transcriptomic profile of *S. aureus *ISP794, we sorted all genes whose transcript levels were ≥ 2-fold upregulated or down-regulated by 10-min up-shifts from 37°C to 43°C or 48°C. The transcript levels of 93 (ca. 4%) of 2410 evaluated genes showed ≥ 2 fold changes at 43°C, among which 39 were down-regulated and 54 upregulated. More extensive changes were recorded at 48°C, since 532 (22%) transcript levels showed ≥ 2 fold changes, with 232 genes being down-regulated and 300 up-regulated. The distributions of the responding genes based on COG functional categories are shown on Additional file 1. Since several COG functional categories included a mixture of annotated and poorly functionally characterized genes (e.g. transcription regulators), we listed all poorly characterized genes in the general function prediction only category (see also Additional file 2).

To provide some indication of basal gene activities under control conditions, we also provided (Additional file 3, 4 and 2) semi-quantitative estimates of normalized signal intensities recorded at 37°C, which were subdivided into four categories (see Methods). Indeed, the highest-intensity signals (75^th ^to 100^th ^percentile) were well correlated with the most abundant transcript products of *S. aureus *predicted to be highly expressed from codon usage [34]. They also correlated quite well with the most abundant proteins revealed by *S. aureus *proteomic studies [35], in particular enzymes involved in DNA, RNA and protein transcription machineries, central metabolism and energy production. Conversely, the lowest intensity signals (25^th ^percentile) recorded at 37°C were contributed by transcripts from poorly expressed genes, such as amino acid biosynthetic pathways known to be repressed by the presence of amino acids in the MHB medium [35].

### Contribution of specific transcriptomic heat stress-responses

As expected from previous studies of heat-shock responses in gram-positive bacteria [13,18,19], all components of *S. aureus *HrcA and CtsR regulons [13] were strongly induced by up-shifts to both 43°C and 48°C (Additional file 3). Transcript levels of the genes regulated by CtsR only (*ctsR*, *mcsA*, *mcsB*, *clpC*, *clpP*, *clpB*) increased by ca. 3–5 fold at 43°C and ca. 3–11 fold at 48°C. We also observed increased expression of genes simultaneously regulated by HrcA and CtsR (*grpE*, *dnaK*, *dnaJ*, *prmA*, *groEL*, *groES*) at both 43°C and 48°C heat-shock. At 48°C, several HSP transcripts were detected at saturating levels by the microarray setting and thus their increased expression was likely under-estimated. To circumvent this problem and also validate the microarray-determined, heat-induced changes, we tested up-regulation of HSP transcript levels by qRT-PCR. Indeed, several gene transcripts (*ctsR*, *mcsA*, *mcsB*, *hrcA*) whose levels were saturated in the microarray scanner after up-shift to 48°C were more highly increased (ca. 6–16-fold) when assayed by qRT-PCR (Additional file 3). In contrast, after bacterial up-shift to 43°C, up-regulation of eight HSP genes (*ctsR*, *mcsA*, *mcsB*, *clpC*, *clpP*, *hrcA*, *dnaK*, and *groEL*) whose levels were not saturated in the microarray scanner, increased to a similar extent by qRT-PCR and microarray. These data confirmed the validity of microarray to quantify changes in bacterial transcript levels.

While the heat-induced upregulation of *ctsR *and *hrCA *may seem paradoxical in view of their previously described repressor activities [13,18] that should down-regulate the transcription of other HSP genes belonging to their respective operons, other parameters may be involved to explain this paradox. First, it has been shown that the CtsR repressor needs ClpC protein to be active [18], and that high temperature may lead to accumulation of conformationally inactive CtsR in the absence of the chaperone co-factor [18]. Second, the global regulatory impact of ClpP protease on *S. aureus *virulence and stress responses also affects the regulation of genes of both the CtsR- and HrcA-controlled regulons [15]. Finally, significant heat shock-induced alterations in energy supplies, which may influence the availability of intracellular ATP levels required for Clp ATPases activities, might also have an impact on the transcriptional control of both CtsR- and HrcA operons.

Finally, to find out whether the presence of a fully functional SigB operon was required for heat-shock transcriptomic responses of HrcA- or/and CtsR-regulated HSP components, we also assayed by qRT-PCR the changes of HSP transcript levels in strain ISPU, a derivative of *S. aureus *strain ISP794 that was genetically restored with a complete *rsbU*^+ ^operon. The 16-fold increase in transcript levels of the SigB-regulated gene *asp23 *confirmed RsbU restoration in the strongly pigmented strain ISPU compared to its non-pigmented RsbU-negative parent ISP794 (data not shown). Additional file 3 shows that heat-induced transcript levels in strain ISPU were either equivalent or <2-fold higher than those recorded in the RsbU-defective parental strain ISP794. Thus, a fully functional SigB operon was not required for induction of heat-shock regulons HrcA and CtsR.

In contrast to those heat-induced gene activities, serine protease HtrA-like (*htrA*) and trigger factor (*tig*) coding genes, as well as several other genes coding for Clp ATPases (*clpL*, *clpQ*, *clpX*, *clpY*) were not at all induced by up-shift to either 43°C or 48°C (Additional file 2), in agreement with previous observations [17,18].

Finally genes coding for in situ repair mechanisms of damaged amino acid residues, such as those belonging to either the methionine sulfoxide reductase complex or the peptidyl-prolyl cis-trans isomerase protein PrsA [11,36], were only marginally up-regulated by temperature up-shifts at 43°C or 48°C (Additional file 2).

### Impact of heat stress on S. aureus growth and survival

**Figure 1:**
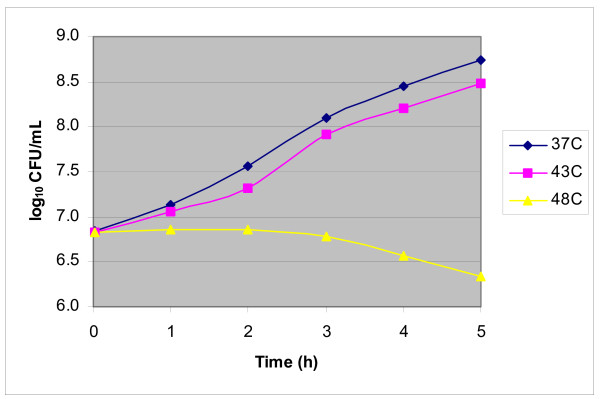
**Comparison of *S. aureus *ISP794 growth rates at 37°C, 43°C, or 48°C**. Viable counts (CFU/ml) of bacterial cultures, grown on Mueller-Hinton broth at the indicated temperatures, were estimated by agar plating of serially diluted samples.

Evaluation of *S. aureus *outcome following temperature up-shifts at 43°C or 48°C was performed by several assays. Both optical density measurements at OD_540 _and viable counts indicated that *S. aureus *cultures were in late-log phase during heat shock. In contrast to OD_540 _profiles that were not influenced by heat stress (data not shown), viable counts revealed that cultures up-shifted to 48°C did not grow further, and, if maintained at the same temperature, showed a slight, ca. 0.3 log_10 _decline in CFU/ml from 60 to 120 min after the heat shock onset. In contrast, CFU counts revealed that *S. aureus *cultures exposed to 43°C or 37°C showed equivalent growth and survival profiles. These data allowed defining 43°C and 48°C as sub-lethal and eventually lethal temperatures, respectively. We also observed that *S. aureus *cultures continually exposed to 43°C or 37°C showed marginally different growth kinetics, while those continuously exposed to 48°C remained growth-arrested at least for a 5 h-period (Figure 1) followed by a significant viability decline at 18 h (data not shown).

To verify that the marginal CFU decline during *S. aureus *heat stress at 48°C did not reflect heat-induced aggregation of the bacterial culture, we also evaluated bacterial viability by fluorescence microscopy, using the Live/Dead BacLight Bacterial Viability assay (see Methods). No significant aggregation was induced by heat exposure and the proportion of propidium iodide-stained, red bacteria increased slowly over time, in agreement with the slowly declining viable counts (data not shown). Finally, the extent of cell lysis was also estimated by the percentage of extracellularly released ATP before and after up-shift from 37°C to 48°C. The results showed nearly equivalent, low contents of extracellular ATP at the different temperatures, which represented <10% of intracellular ATP assayed in parallel and confirmed the marginal cell lysis (data not shown).

### Additional heat-stress transcriptomic responses from various metabolic pathways

A large proportion of genes whose transcript levels showed ≥ 2-fold changes after up-shifts to either 43°C or 48°C belonged either to additional stress response pathways that were not regulated by CtsR-, HrcA-, and/or SigB, or to major metabolic pathways that likely contributed to the physiological adjustment and survival of heat-stress exposed bacteria. The Additional file 4 shows selected examples of up- or down-regulated genes representative of the different metabolic categories. A more exhaustive list of relevant gene transcripts and pathways is presented in the Additional file 2, which also includes altered genes of general function prediction only or unknown function.

#### Regulation of osmotic balance

Some heat-induced genes likely contributed to osmotolerance, such as those encoding glycine betaine transporter (*opuD*), choline dehydrogenase (*betA*), and glycine aldehyde dehydrogenase (*gbsA*). In contrast, transcript levels of the high affinity proline permease (*putP*) and proline betaine transporter (*proP*) remained constant, while those encoding the glycine betaine/carnitine/choline ABC transporter operon (*opuCA/CB/CD*), or the potassium-ABC transporter operon (*kdpABC*) and its regulator *kdpDE*, were down-regulated.

#### Oxidative stress responses

Some transcripts up-regulated by temperature up-shift at 48°C but not at 43°C were coding for enzymes coping with oxidative stress, in particular the superoxide dismutase gene *sodA*, and to a lesser extent (ratio: 1.84) thioredoxin (*trxA*) but not thioredoxin reductase (*trxB*). Occurrence of a heat-induced DNA damage at 48°C but not 43°C, potentially linked with oxidative stress, was suggested by increased transcript levels of nine genes coding for enzymes involved in DNA repair or/and recombination, namely *dinB*, *uvrC*, *addA*, *recU, mutS2*, the transcription-repair coupling factor *mfd*, the exonuclease SbcC, a zinc-dependent DNA glycosylase (SA1512), and to a lower extent *polA *encoding DNA polymerase I (ratio: 1.84). Part of those genes coding for DNA-damage repair and recombination enzymes were previously reported to be up-regulated, though to a variable extent, by *S. aureus *exposure to DNA-damaging agents such as mitomycin C [33] and ciprofloxacin [37], low pH [38], nitrite stress [39], peracetic acid [40] and cell-wall-active antibiotics [36]. In contrast, only one (*uvrC*) DNA-damage repair gene was up-regulated in *S. aureus *up-shifted to 43°C for 30 min [33]. In contrast to cell exposed to DNA-damaging agents [33,37], we did not observe up-regulation of *recA *and *lexA *genes at 43°C or 48°C, which indicated the lack of a significant SOS response in heat-stressed bacteria.

#### Metal transporters

Several genes coding for influx or efflux metal transporters showed altered activities, which indicated possible dysregulation of metal homeostasis by temperature up-shifts. Except for the up-regulation of *nixA *coding for a high affinity nickel uptake transporter that seemed to be linked with urea cycle activation (see below), other up-regulated genes were encoding copper (*copA*) and zinc (*czrAB*) efflux transporters. Despite extensive studies, we lack a global, comprehensive model describing the regulation of physiological, intracellular levels of iron and other heavy metals in *S. aureus*, under normal and stressful conditions [41,42]. While the peroxide operon regulator PerR was up-regulated at both 48°C and 43°C, transcript levels of some but not all PerR-regulated genes, such as *katA *(catalase), *fnt *(ferritin), and *dps*/*mgrA *also showed some increase at 48°C (see Additional file 2). The down-regulation of ABC transporter genes for other metallic cations such as manganese (*mntABC*) or cobalt might also indicate the need to avoid intracellular accumulation of potentially toxic levels of free heavy metals at 48°C.

### Adjustment of ATP-providing pathways in heat-shocked S. aureus

Increasing, heat-triggered demand for protein- and DNA-repair mechanisms leads to higher consumption of cellular energy resources. Interestingly, nearly identical intracellular ATP levels were assayed in bacterial cultures shifted to 43°C (487 ± 7 pM/10^9 ^CFU), 48°C (483 ± 27 pM/10^9 ^CFU), or left at 37°C (469 ± 60 pM/10^9 ^CFU). Two previous reports also mentioned that heat stress did not decrease, but could even transiently increase, ATP levels in *S. aureus *[23] or *E. coli *[43]. To understand how heat-shocked bacteria could maintain constant intracellular ATP levels despite increased needs for repair systems, we evaluated gene expression changes in major energy-providing, metabolic pathways. Expression of genes encoding components of the glycolytic pathway remained quite constant after up-shifts to 43°C and 48°C, except for a nearly significant 2-fold decline of enolase (*eno*) at 48°C (see Additional file 2). More contrasting data were obtained with expression of TCA cycle genes, with three of them, namely *citZ *(citrate synthase), *citC *(isocitrate dehydrogenase), and *odhB *(dihydrolipoamide succinyltransferase), being up-regulated by heat-shock (48°C), while *citB *coding for the key TCA regulatory component aconitase was down-regulated [44]. It is unclear whether increased expression of *citZ*, *citC*, and *odhB*, which are conflicting with down-regulation of the TCA regulator aconitase, indicates an overall increased activity of the TCA cycle, or reflects individual contributions of some TCA components to other pathways. Indeed, citrate synthase may contribute to gluconeogenesis (by shuttling citrate to oxaloacetate and back to pyruvate/phosphoenolpyruvate) and dihydrolipoamide succinyltransferase to lysine degradation. Other microarray studies also reported induction of some TCA cycle components in stress-exposed *S. aureus *[37,38]. Moreover, increased transcription at 48°C of *zwf *(glucose 6 phosphate dehydrogenase) and *pycA *(pyruvate carboxylase) also suggested activation of the pentose phosphate and gluconeogenesis pathways, respectively (Additional file 4).

We also noticed increased transcription at 48°C of three key enzymes (*thiE*, *thiM*, *thiD*) involved in the biosynthetic pathway leading to thiamine pyrophosphate coenzyme (ThPP), involved in major decarboxylation reactions of glycolysis, TCA and pentose phosphate pathways. A similar up-regulation of three key enzymes (*ribA*, *ribB*, *ribD*) coding for riboflavin synthesis was observed at 48°C. Both ThPP and FAD are also important for branched-chain fatty acid biosynthesis, derived from the catabolism of the branched-chain amino acids leucine, valine, and isoleucine [45,46]. Moreover, increased expression of ThPP is also essential for biosynthesis of branched amino acids, and fit well with microarray data indicating derepression of 3 genes (*leuA*, *leuB*, *leuC*) coding for leucine biosynthesis. Adjustment of branched-chain fatty acid biosynthesis may be an important defense mechanism against heat-induced membrane disordering and contribute to restoring optimal membrane fluidity and proton impermeability [47] (see below).

### Analysis of key metabolites in *S. aureus *growth medium

To evaluate the physiological significance of our transcriptomic data, we analyzed in parallel the presence of key metabolites in the growth medium, before and after temperature up-shifts. Freshly prepared MHB, before bacterial inoculation, contained relatively low levels of free glucose (0.38 mM), which were rapidly depleted (<0.001 mM) during the pre-shock growth period, as found in other studies [48,49]. Extracellular starch levels, an abundant component of MHB, which was looked as a potential glucose-providing source, remained absolutely constant (assayed as 1.2–1.3 mg/ml of glucose equivalent) throughout bacterial growth. This suggested that *S. aureus *could not use starch as a nutrient source presumably because of the lack of extracellular amylolytic activity. Collectively, our transcriptomic and physiological data strongly indicated that, after glucose exhaustion from the medium, *S. aureus *was forced to use the most abundant alternative carbon sources that were amino acid or peptide mixtures provided in the casein acid hydrolysate component of MHB. Recent metabolic studies indicate that the catabolism of several amino acids can feed both TCA cycle and gluconeogenesis pathways by producing essential intermediates oxaloacetate, oxoglutarate, phosphoenolpyruvate, and pyruvate [44,49,50]. These metabolic studies also indicate that glucose depletion leads to derepression of TCA cycle components [44], as confirmed by our transcriptomic data showing their high expression levels at 37°C. While significant levels (3.0–3.5 mM) of acetate were detected in MHB just before and after temperature up-shifts, these levels remained marginal compared to those (ca. 15–20 mM) recorded in other studies [44,48,51], and were not sufficient to significantly acidify the growth medium. In contrast to gene activities of the glycolytic, pentose phosphate shunt, and TCA cycle pathways, most nitrate/nitrite reductase components were down-regulated at both 43°C and 48°C. Furthermore, several major fermentative pathway components were markedly down-regulated by heat stress at both 43°C and 48°C, in particular alcohol (*adhE*, *adh1*), lactate (*ldhA*, *ldhB*) and formate (*fdh*) dehydrogenases. Biochemical assays confirmed the marginal levels of L-lactate (0.3–0.5 mM) and D-lactate (< 0.15 mM) in MHB. The down-regulation of energy-providing fermentative pathways suggests that they may be energetically less efficient for heat-exposed *S. aureus*.

### Adjustment of ATP-consuming pathways in heat-shocked S. aureus

Two categories of ATP-requiring biosynthetic pathways showed a significant, global reduction in transcript levels. The first category included the purine and pyrimidine synthetic pathways whose fifteen and nine components, respectively, were down-regulated to the same extent (Additional files 4 and 2). In contrast, transcript levels of *drm *(phosphopentomutase) and *pnp *(purine nucleoside phosphorylase), coding for salvage pathways, were markedly increased. Induction of salvage pathways contributes to the recycling of bases, by diminishing the high energetic costs of their multistep re-synthesis, and may also provide ribose to the pentose phosphate cycle. The second category of down-regulated transcript levels at 48°C included genes coding for 13 amino acyl-tRNA synthetases, among which eight were also decreased at 43°C (Additional files 4 and 2). Conversely, expression of cysteinyl-tRNA synthetase was significantly increased at 48°C.

In contrast, expression of most other genes coding for major biosynthetic apparatus of replication, transcription, and translation, e.g. ribosomal proteins, DNA or RNA synthesis, was not or only marginally affected by heat shock (see Additional file 2), except for *rnc *coding for RNase III whose expression was up-regulated at both 43°C and 48°C. A similar situation prevailed among cell wall and membrane biogenesis components, with only 10% of altered transcripts, in contrast to autolytic components whose expression was more affected by heat shock. Among cell division-regulating components, only *scdA *transcript levels, coding for a cell division and morphogenesis-related protein, were specifically reduced at both 43°C and 48°C.

Another category of ATP-consuming activities, whose expression appeared down-regulated, included 13 out of 15 evaluated ATP-dependent components of amino acid or peptide transporters (Additional files 4 and 2). Microarray data confirmed that amino acid/oligopeptide, transport was essential to cell metabolism because most amino acid synthetic pathways were repressed at 37°C. However, some of those amino acid pathways were strongly induced by up-shift to 48°C, as revealed by increased transcript levels (2.5–18 fold) of biosynthetic enzymes for lysine, tryptophan, glutamate, histidine, and branched chain amino acids. Up-regulation of those amino acid synthetic pathways, despite being high consumers of ATP, might indicate an increasing need of some amino acids during heat stress, possibly amplified by a decreased efficiency of some amino acid, ATP-driven transport systems. Of note, the content of free amino acids in MHB remained abundant throughout bacterial growth as well as after heat shock exposure (data not shown), which ruled out a specific depletion of some amino acids as observed in a previous study [49]. Therefore, the marginal decline in extracellular amino acid supply was not sufficient for explaining the selective, biosynthetic induction of some amino acids during heat stress at 48°C.

Since transcriptomic data suggest a decreased efficiency of energy-dependent transport systems in heat stressed-bacteria, this observation can be supported by the documented effects of increased temperature on bacterial membrane fluidity, which are known to alter proton impermeability and the proton-motive force [47,52]. These heat-induced alterations in the membrane physico-chemical properties may require changes in its lipid composition for fluidity adjustment [47,52].

### Summary of transcriptomic and metabolic observations into a relevant metabolic model

Transcriptomic and metabolic data of *S. aureus *exposed to a sub-lethal (43°C) or eventually lethal (48°C) temperature can be summarized as follows: (i) heat stress exposure generates an increased ATP demand for protein- and DNA-repair; (ii) constant intracellular levels of ATP could be maintained despite a relative decline of ATP-generating sources, in particular fermentation and microaerophilic nitrate and nitrite reduction pathways. (iii) exhaustion of glucose supply during *S. aureus *culture preceding heat shock force the bacteria to feed ATP-generating pathways with amino acids metabolized into oxoglutarate, oxaloacetate, phosphoenolpyruvate and pyruvate, as essential TCA cycle and gluconeogenesis intermediates. We can further speculate that the decreased expression of a vast majority of amino acyl-tRNA synthetases might promote the release of amino acids that feed energy-providing pathways, though this may eventually compromise protein synthesis during prolonged heat shock.

**Figure 2:**
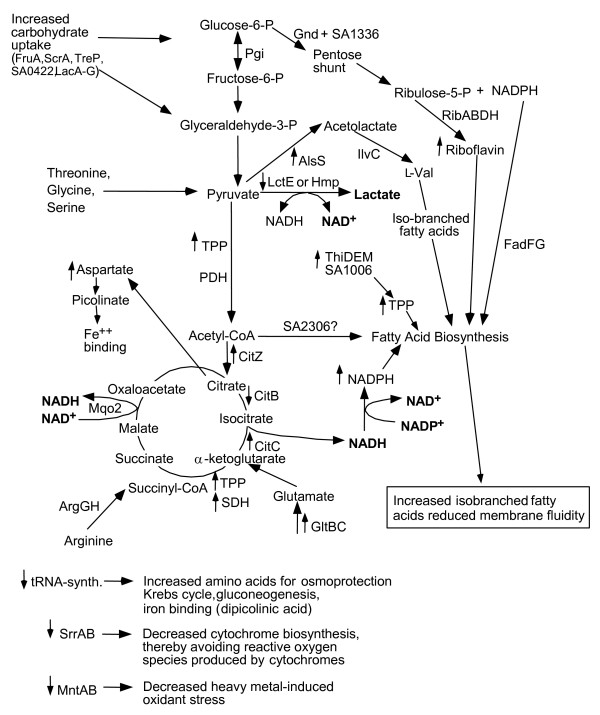
**Schematic representation of the major metabolic pathways that are up- or down-regulated by heat stress at 48°C**. The three letter designations for the enzymes involved in the heat stress response can be found in the KEGG web site for *S. aureus *N135 http://www.genome.jp/kegg/. When there are several genes within the same operon that are increased, then the three letter designation is followed by capital letters, which represents the different enzymes (genes). When the gene/enzyme is not definitively known, then the gene number in the N315 ordered sequence tag numbering is given, e.g., SA1336 for glucose-6-phosphate 1-dehydrogenase. Arrows before the enzymes indicate significant increases (upward arrow) or decreases (downward arrow) in the transcripts or the key metabolic product that is produced by the pathway. All of the steps in the metabolic pathways are not shown, rather just key branch-points, so as to simplify the figure. Several important changes are shown at the bottom of the slide that do not fit into the central metabolism of the cell.

The metabolic model proposed below (Figure 2) attempts to integrate metabolic responses (including already mentioned protein and DNA-repair pathways) of heat-stressed *S. aureus *with the predictable, heat-induced membrane disordering, in which increased motion of the lipid molecules may lead to increased proton transmembrane permeability and potentially severe bioenergetic consequences [47]. Studies in different bacterial species indicate that optimal membrane fluidity and proton impermeability can be restored by adjustment of its fatty acid composition [47,52]. Major lipid biosynthetic pathways require high levels of NADPH and acetyl-CoA, which may explain up-regulation of the pentose phosphate cycle during heat shock. This may be further supported by up-regulation of ThPP and FAD biosynthetic pathways that are essential cofactors for biosynthesis of branched amino acids, whose catabolites are important precursors of branched-chain fatty acid biosynthesis [45,46]. More detailed experimental studies are needed to confirm the importance of these adaptive mechanisms in *S. aureus*. Finally, the metabolic model also integrates the necessity for heat-stressed *S. aureus *to down-regulate the production of reactive oxygen species that may be generated *via *electron transport-generated ATP, in particular by reducing levels of free metals, such as iron, that may promote generation of superoxide and hydroxyl radicals [41,42,53].

## Conclusion

Combined molecular and biochemical approaches are required for a deeper understanding of mechanisms of ATP homeostasis in *S. aureus *and analyze its impact on the loss of replicative functions and viability during exposure to high temperatures as well as other stressing conditions. This experimental approach should also contribute to the discovery of new antimicrobial targets and development of innovative anti-infective strategies.

## Methods

### Strains and growth conditions

*S. aureus *strain ISP794 (NCTC8325) was used for most experiments. Strain ISPU is a derivative of ISP794 whose SigB functional activity was restored by transduction with a phage lysate prepared from the *rsbU*^+^-restored strain GP268, as described [54]. Strain ISPU yields strongly pigmented colonies on Mueller-Hinton agar (MHA), and its genotype was verified by a PCR assay [54].

*S. aureus *strains were routinely grown without shaking in Mueller-Hinton broth (MHB; Beckton Dickinson). For protocols evaluating *S. aureus *transcriptional responses at different temperatures, bacterial cultures were prepared by growing 100-fold dilutions of overnight cultures in 15 ml MHB for 5 h at 37°C, to an OD_540 _of 0.6 corresponding to 2–4 × 10^8 ^CFU/ml. These bacterial culture conditions have been used for several previous studies of *S. aureus *virulence (adhesins, toxins, gene expression) [54-57] in vitro and for experimental infections in animal models [58,59], as well as for antibiotic susceptibility testing assays [60]. Then, the 5-h pre-cultures were transferred either to 43°C or 48°C or left at 37°C for 10 min. Immediately after the heat shock, all cultures were directly transferred to RNAprotect Bacteria Reagent (Qiagen).

### Total RNA extraction and labeling

We followed a previously described procedure with slight modifications [57]. Following mixing with 30-ml RNAprotect reagent and incubation at room temperature for 15 min, each culture was centrifuged for 15 min at 5000 r.p.m. at 4°C. Bacterial pellets were suspended in PBS and treated with 200 μg/ml lysostaphin at 37°C for 10 min. RNA was purified using the RNeasy extraction kit (Qiagen), then treated with DNAse, and the absence of contaminating DNA verified by PCR. Purified RNA samples were analyzed using the RNA NanoLab chip on the 2100 Bioanalyser (Agilent).

### DNA microarray

The major characteristics of our customized StaphChip oligoarray, used for transcriptomic profiling or comparative genome hybridization, have been previously described [57,61-64]. The oligoarray version used in this study included 8'436 40- to 60-mer probes, recognizing >99% of ORFs of *S. aureus *N315, Mu50, COL, MW2, MRSA252, and MSSA476 genomes, plus those of the four plasmids pN315, pVRSA, pT181, pSAS. Total RNAs (10 μg) from heat-exposed and control strains were labeled in parallel with Cy3-dCTP and Cy5-dCTP, then purified as described [57]. For competitive hybridization using a dual-labeled experimental approach, equivalent amounts (ca. 6 μg/ml) of Cy3-labelled and Cy5-labelled cDNAs were diluted in 115 μl Agilent hybridization buffer and cohybridized for 17 h at 60°C. Slides were washed and dried under nitrogen flow as described [61].

Slides were scanned (Agilent) using 100% photomultiplier tube power for both wavelengths as described [61]. All positive and significant local-background-subtracted signals, obtained using Feature Extraction software (version 7.5, Agilent), were corrected for unequal dye incorporation or unequal load of the labeled product. The algorithm consisted of a rank consistency filter and a curve fit using the default LOWESS (locally weighted linear regression) method. Irregular or saturated spots, as well as spots showing a reference signal lower than background plus two standard deviations were excluded from subsequent analysis [57,61].

All Feature Extraction-processed dye-normalized signals from the oligoarray were subdivided into four categories, as previously described [57], according to their intensities in control conditions at 37°C: the 25^th ^percentile of probes yielding the lower-intensity signals (24 to 512 units), followed by the 25^th ^to 50^th ^percentile (513 to 1655 units), the 50^th ^to 75^th ^percentile (1656 to 4543 units) and the 75^th ^to 100^th ^percentile, yielding the highest-intensity signals (4544 to 89900 units).

We previously demonstrated that for most assayed genes, changes in transcript levels, expressed as ratios of red to green signal intensities, were highly reproducible on multiple probes recognizing non-overlapping regions of each transcript[57]. Accordingly, a minority of transcripts that showed widely different ratios from multiple probes were excluded. For all other genes whose signal ratios, recorded from multiple probe subsets, were closely related and consistently ≥ 2 or ≤ 0.5, the mean signal ratio of each relevant transcript was first determined for each daily experiment. Finally, the overall mean (± SEM) ratio was evaluated for each relevant gene from three independent biological replicates, and each transcript whose ratio was ≥ 2 or ≤ 0.5, and statistically validated by *t*-test at a *P *level of 0.05, was considered as differentially expressed [57]. Since experiments evaluating transcriptomic changes from 37°C to 43°C or 48°C was performed on different days, no variance analysis of transcriptomic changes recorded at all three temperatures was performed.

Processed microarray data files have been deposited in the Gene Expression Omnibus (GEO) database http://www.ncbi.nlm.nih.gov/projects/geo under accession number GSE12920. Gene designations, predicted functions, and functional categorization were derived from NCBI and SwissProt-Expasy updated databases of completed *S. aureus*. For convenience, we used ORF numbers from *S. aureus *strain N315, except when indicated. Comparison of our microarray data with those of other *S. aureus *transcriptomic studies was facilitated by the use of the SAMMD microarray meta-database [65]http://bioinformatics.org/sammd/main.htm.

### Real-time quantitative RT-PCR

mRNA levels of a subset of selected genes were determined by quantitative reverse transcriptase PCR (qRT-PCR) using the one-step reverse transcriptase qPCR Master Mix kit (Eurogentec), as described previously [56]. All primers and probes are listed in the Additional file 5 and were designed using PrimerExpress Software (version 3.0); Applied Biosystem) and obtained from Eurogentec or Invitrogen. Conditions for reverse transcription, PCR, detection of fluorescence emission, and normalization of the mRNA levels of the target genes on the basis of their 16S rRNA levels were described previously [56,66]. qRT-PCR data represent the mean (± SEM) of three independent, biological replicates. The statistical significance of temperature-specific differences in normalized cycle threshold values for each transcript was evaluated by paired *t*-test, and data were considered significant when *P *was < 0.05.

### Evaluation of growth kinetics, survival, and cell lysis of S. aureus at different temperatures

Four different techniques were used: (i) optical density measurements at OD_540_; (ii) viable counts (CFU/ml) estimates of serially diluted cultures; (iii) staining of the bacteria using the Live/Dead BacLight Bacterial Viability kit L7007 (Invitrogen) following the manufacturer's instructions; (iv) the extent of cell lysis was also estimated by the percentage of extracellularly released ATP (see below).

### Measurement of ATP levels

In initial studies, cultures were sampled at appropriate time points, then centrifuged and resuspended in 1 ml fresh MHB. In parallel, supernatants were filter-sterilized and transferred into new tubes. Alternatively, ATP levels were also directly assayed in non-centrifuged cultures. Intracellular as well as extracellular ATP levels were recorded with BacTiter-Glo™ kit from Promega, following the manufacturer's instructions. The reaction mixture contained 100 μl of serially diluted bacterial extracts or filter-sterilized, culture supernatants, which were mixed with 100 μl of the BacTiter-Glo reagent, in white, 96 well plates (Microlite™ TCT, Promega). Each sample was assayed in triplicate wells, and luminescence was detected by fluorometry (LumiCount^TR^, Packard Instrument). Results from three independent biological replicates were expressed in nanomolar units according to standard curves generated with purified ATP (Sigma).

### Determination of glucose, starch, L- and D-lactate, and acetate levels in culture supernatants

To monitor metabolic parameters influenced by different thermal conditions, we assayed at appropriate time points glucose, acetate, L- and D-lactate levels in filter-sterilized, culture supernatants, using kits purchased from R-Biopharm AG according to manufacturer's instructions. We also assayed the glucose, acetate, and L-/D-lactate contents of fresh, sterile MHB medium, whose detailed composition is not available. Of note, we also performed a time-course of the starch levels of MHB during bacterial growth, using a commercial kit of R-Biopharm, to determine whether it might provide a nutrient source for *S. aureus*. Results from three independent biological replicates were expressed in molar units of glucose equivalents

## Competing interests

The authors declare that they have no competing interests.

## Authors' contributions

PV, BF, WLK, and DL were involved in the study design. BF performed the experimental study and acquisition of data. BF and PV performed data analysis and wrote the final draft of this paper. FG, RAP, and DL provided input into subsequent drafts and iteration of this manuscript. All authors read and approved the final manuscript.

## Supplementary Material

Additional file 1 COG function categories of genes whose transcript levels showed >2-fold changes after 10 minute heat shock. Click here for file

Additional file 2 **Functional categories of S. aureus genes up-regulated, down-regulated, or not significantly (<2-fold) changed, by 10 min heat shock**. Exhaustive list of relevant gene transcripts and pathways. Click here for file

Additional file 3 Evaluation by micro array and qRT-PCR of the transcriptiopnal responses of S aureus heat stress regulons. Click here for file

Additional file 4 **Selected examples of S. aureus genes up-regulated, down-regulated, or not significantly (<2-fold) changed, by 10 min heat shock**. Selected examples of up- or down-regulated genes representative of the different metabolic categories. Click here for file

Additional file 5 Sequences of primers and TaqMan probes used in this study. Click here for file

## References

[B1] Lowy FD *Staphylococcus aureus *infections N Engl J Med 1998 339 520 532 10.1056/NEJM199808203390806 9709046

[B2] Furuya EY Lowy FD Antimicrobial-resistant bacteria in the community setting Nat Rev Microbiol 2006 4 36 45 10.1038/nrmicro1325 16357859

[B3] Sanford MD Widmer AF Bale MJ Jones RN Wenzel RP Efficient detection and long-term persistence of the carriage of methicillin-resistant *Staphylococcus aureus* Clin Infect Dis 1994 19 1123 1128 7888543 10.1093/clinids/19.6.1123

[B4] Kluytmans JA Van Belkum A Verbrugh H Nasal carriage of *Staphylococcus aureus*: Epidemiology, underlying mechanisms, and associated risks Clin Microbiol Rev 1997 10 505 520 9227864 10.1128/cmr.10.3.505 PMC172932

[B5] Gill SR Fouts DE Archer GL Mongodin EF Deboy RT Ravel J Paulsen IT Kolonay JF Brinkac L Beanan M Dodson RJ Daugherty SC Madupu R Angiuoli SV Durkin AS Haft DH Vamathevan J Khouri H Utterback T Lee C Insights on evolution of virulence and resistance from the complete genome analysis of an early methicillin-resistant *Staphylococcus aureus *strain and a biofilm-producing methicillin-resistant *Staphylococcus epidermidis *strain J Bacteriol 2005 187 2426 2438 15774886 10.1128/JB.187.7.2426-2438.2005 PMC1065214

[B6] Novick RP Autoinduction and signal transduction in the regulation of staphylococcal virulence Mol Microbiol 2003 48 1429 1449 10.1046/j.1365-2958.2003.03526.x 12791129

[B7] Blevins JS Gillaspy AF Rechtin TM Hurlburt BK Smeltzer MS The staphylococcal accessory regulator (*sar*) represses transcription of the *Staphylococcus aureus *collagen adhesin gene (*cna*) in an *agr*-independent manner Mol Microbiol 1999 33 317 326 10.1046/j.1365-2958.1999.01475.x 10411748

[B8] Kuroda M Ohta T Uchiyama I Baba T Yuzawa H Kobayashi I Cui L Oguchi A Aoki K Nagai Y Lian J Ito T Kanamori M Matsumaru H Maruyama A Murakami H Hosoyama A Mizutani-Ui Y Takahashi NK Sawano T Whole genome sequencing of meticillin-resistant *Staphylococcus aureus* Lancet 2001 357 1225 1240 10.1016/S0140-6736(00)04403-2 11418146

[B9] Cheung AL Bayer AS Zhang G Gresham H Xiong YQ Regulation of virulence determinants in vitro and in vivo in *Staphylococcus aureus* FEMS Immunol Med Microbiol 2004 40 1 9 10.1016/S0928-8244(03)00309-2 14734180

[B10] Clements MO Foster SJ Stress resistance in *Staphylococcus aureus* Trends Microbiol 1999 7 458 462 10.1016/S0966-842X(99)01607-8 10542426

[B11] Visick JE Clarke S Repair, refold, recycle: how bacteria can deal with spontaneous and environmental damage to proteins Mol Microbiol 1995 16 835 845 10.1111/j.1365-2958.1995.tb02311.x 7476182

[B12] Gottesman S Wickner S Maurizi MR Protein quality control: triage by chaperones and proteases Genes Dev 1997 11 815 823 10.1101/gad.11.7.815 9106654

[B13] Chastanet A Fert J Msadek T Comparative genomics reveal novel heat shock regulatory mechanisms in *Staphylococcus aureus *and other Gram-positive bacteria Mol Microbiol 2003 47 1061 1073 10.1046/j.1365-2958.2003.03355.x 12581359

[B14] Singh VK Utaida S Jackson LS Jayaswal RK Wilkinson BJ Chamberlain NR Role for dnaK locus in tolerance of multiple stresses in *Staphylococcus aureus* Microbiology 2007 153 3162 3173 10.1099/mic.0.2007/009506-0 17768259

[B15] Michel A Agerer F Hauck CR Herrmann M Ullrich J Hacker J Ohlsen K Global regulatory impact of ClpP protease of *Staphylococcus aureus *on regulons involved in virulence, oxidative stress response, autolysis, and DNA repair J Bacteriol 2006 188 5783 5796 16885446 10.1128/JB.00074-06 PMC1540084

[B16] Chatterjee I Becker P Grundmeier M Bischoff M Somerville GA Peters G Sinha B Harraghy N Proctor RA Herrmann M *Staphylococcus aureus *ClpC is required for stress resistance, aconitase activity, growth recovery, and death J Bacteriol 2005 187 4488 4496 15968059 10.1128/JB.187.13.4488-4496.2005 PMC1151783

[B17] Frees D Qazi SN Hill PJ Ingmer H Alternative roles of ClpX and ClpP in *Staphylococcus aureus *stress tolerance and virulence Mol Microbiol 2003 48 1565 1578 10.1046/j.1365-2958.2003.03524.x 12791139

[B18] Frees D Chastanet A Qazi S Sorensen K Hill P Msadek T Ingmer H Clp ATPases are required for stress tolerance, intracellular replication and biofilm formation in *Staphylococcus aureus* Mol Microbiol 2004 54 1445 1462 10.1111/j.1365-2958.2004.04368.x 15554981

[B19] Derre I Rapoport G Msadek T CtsR, a novel regulator of stress and heat shock response, controls *clp *and molecular chaperone gene expression in gram-positive bacteria Mol Microbiol 1999 31 117 131 10.1046/j.1365-2958.1999.01152.x 9987115

[B20] Wu SW De Lencastre H Tomasz A Sigma-B, a putative operon encoding alternate sigma factor of *Staphylococcus aureus *RNA polymerase: Molecular cloning and DNA sequencing J Bacteriol 1996 178 6036 6042 8830703 10.1128/jb.178.20.6036-6042.1996 PMC178463

[B21] Kullik I Giachino P The alternative sigma factor σ^B ^in *Staphylococcus aureus*: Regulation of the sigB operon in response to growth phase and heat shock Arch Microbiol 1997 167 151 159 10.1007/s002030050428 9042755

[B22] Bischoff M Dunman P Kormanec J Macapagal D Murphy E Mounts W Berger-Bachi B Projan S Microarray-based analysis of the *Staphylococcus aureus *sigmaB regulon J Bacteriol 2004 186 4085 4099 15205410 10.1128/JB.186.13.4085-4099.2004 PMC421609

[B23] Pane-Farre J Jonas B Forstner K Engelmann S Hecker M The sigma(B) regulon in *Staphylococcus aureus *and its regulation Int J Med Microbiol 2006 296 237 258 10.1016/j.ijmm.2005.11.011 16644280

[B24] Gertz S Engelmann S Schmid R Ziebandt AK Tischer K Scharf C Hacker J Hecker M Characterization of the σ^B ^regulon in *Staphylococcus aureus* J Bacteriol 2000 182 6983 6991 11092859 10.1128/JB.182.24.6983-6991.2000 PMC94824

[B25] Senn MM Giachino P Homerova D Steinhuber A Strassner J Kormanec J Fluckiger U Berger-Bachi B Bischoff M Molecular analysis and organization of the sigmaB operon in *Staphylococcus aureus* J Bacteriol 2005 187 8006 8019 16291674 10.1128/JB.187.23.8006-8019.2005 PMC1291286

[B26] Gertz S Engelmann S Schmid R Ohlsen K Hacker J Hecker M Regulation of σ^B^-dependent transcription of *sigB *and *asp23 *in two different *Staphylococcus aureus *strains Mol Gen Genet 1999 261 558 566 10.1007/s004380051001 10323238

[B27] Giachino P Engelmann S Bischoff M σ^B ^activity depends on RsbU in *Staphylococcus aureus* J Bacteriol 2001 183 1843 1852 11222581 10.1128/JB.183.6.1843-1852.2001 PMC95078

[B28] Bischoff M Entenza JM Giachino P Influence of a functional *sigB *operon on the global regulators *sar *and *agr *in *Staphylococcus aureus* J Bacteriol 2001 183 5171 5179 11489871 10.1128/JB.183.17.5171-5179.2001 PMC95394

[B29] Palma M Cheung AL sigma(B) activity in *Staphylococcus aureus *is controlled by RsbU and an additional factor(s) during bacterial growth Infect Immun 2001 69 7858 7865 11705968 10.1128/IAI.69.12.7858-7865.2001 PMC98882

[B30] Iandolo JJ Ordal ZJ Repair of thermal injury of *Staphylococcus aureus* J Bacteriol 1966 91 134 142 5903089 10.1128/jb.91.1.134-142.1966 PMC315922

[B31] Bucker ER Martin SE Effect of free-radical scavengers on enumeration of thermally stressed cells of *Staphylococcus aureus *MF-31 Appl Environ Microbiol 1982 43 1020 1025 6285821 10.1128/aem.43.5.1020-1025.1982 PMC244180

[B32] Bucker ER Martin SE Superoxide dismutase activity in thermally stressed *Staphylococcus aureus* Appl Environ Microbiol 1981 41 449 454 7235693 10.1128/aem.41.2.449-454.1981 PMC243714

[B33] Anderson KL Roberts C Disz T Vonstein V Hwang K Overbeek R Olson PD Projan SJ Dunman PM Characterization of the *Staphylococcus aureus *heat shock, cold shock, stringent, and SOS responses and their effects on log-phase mRNA turnover J Bacteriol 2006 188 6739 6756 16980476 10.1128/JB.00609-06 PMC1595530

[B34] Karlin S Theriot J Mrazek J Comparative analysis of gene expression among low G+C gram-positive genomes Proc Natl Acad Sci USA 2004 101 6182 6187 15069198 10.1073/pnas.0401504101 PMC395943

[B35] Kohler C Wolff S Albrecht D Fuchs S Becher D Buttner K Engelmann S Hecker M Proteome analyses of *Staphylococcus aureus *in growing and non-growing cells: a physiological approach Int J Med Microbiol 2005 295 547 565 10.1016/j.ijmm.2005.08.002 16325551

[B36] Utaida S Dunman PM Macapagal D Murphy E Projan SJ Singh VK Jayaswal RK Wilkinson BJ Genome-wide transcriptional profiling of the response of *Staphylococcus aureus *to cell-wall-active antibiotics reveals a cell-wall-stress stimulon Microbiology 2003 149 2719 2732 10.1099/mic.0.26426-0 14523105

[B37] Cirz RT Jones MB Gingles NA Minogue TD Jarrahi B Peterson SN Romesberg FE Complete and SOS-mediated response of *Staphylococcus aureus *to the antibiotic ciprofloxacin J Bacteriol 2007 189 531 539 17085555 10.1128/JB.01464-06 PMC1797410

[B38] Bore E Langsrud S Langsrud O Rode TM Holck A Acid-shock responses in *Staphylococcus aureus *investigated by global gene expression analysis Microbiology 2007 153 2289 2303 10.1099/mic.0.2007/005942-0 17600073

[B39] Schlag S Nerz C Birkenstock TA Altenberend F Gotz F Inhibition of staphylococcal biofilm formation by nitrite J Bacteriol 2007 189 7911 7919 17720780 10.1128/JB.00598-07 PMC2168742

[B40] Chang W Toghrol F Bentley WE Toxicogenomic response of *Staphylococcus aureus *to peracetic acid Environ Sci Technol 2006 40 5124 5131 10.1021/es060354b 16955917

[B41] Horsburgh MJ Clements MO Crossley H Ingham E Foster SJ PerR controls oxidative stress resistance and iron storage proteins and is required for virulence in *Staphylococcus aureus* Infect Immun 2001 69 3744 3754 11349039 10.1128/IAI.69.6.3744-3754.2001 PMC98383

[B42] Horsburgh MJ Ingham E Foster SJ In *Staphylococcus aureus*, Fur is an interactive regulator with PerR, contributes to virulence, and is necessary for oxidative stress resistance through positive regulation of catalase and iron homeostasis J Bacteriol 2001 183 468 475 11133939 10.1128/JB.183.2.468-475.2001 PMC94901

[B43] Soini J Falschlehner C Mayer C Bohm D Weinel S Panula J Vasala A Neubauer P Transient increase of ATP as a response to temperature up-shift in *Escherichia coli* Microb Cell Fact 2005 4 9 15804347 10.1186/1475-2859-4-9 PMC1087501

[B44] Somerville GA Chaussee MS Morgan CI Fitzgerald JR Dorward DW Reitzer LJ Musser JM *Staphylococcus aureus *aconitase inactivation unexpectedly inhibits post-exponential-phase growth and enhances stationary-phase survival Infect Immun 2002 70 6373 6382 12379717 10.1128/IAI.70.11.6373-6382.2002 PMC130419

[B45] Beck HC Hansen AM Lauritsen FR Catabolism of leucine to branched-chain fatty acids in *Staphylococcus xylosus* J Appl Microbiol 2004 96 1185 1193 10.1111/j.1365-2672.2004.02253.x 15078537

[B46] Beck HC Branched-chain fatty acid biosynthesis in a branched-chain amino acid aminotransferase mutant of *Staphylococcus carnosus* FEMS Microbiol Lett 2005 243 37 44 10.1016/j.femsle.2004.11.041 15667998

[B47] Konings WN Albers SV Koning S Driessen AJ The cell membrane plays a crucial role in survival of bacteria and archaea in extreme environments Antonie Van Leeuwenhoek 2002 81 61 72 10.1023/A:1020573408652 12448706

[B48] Somerville GA Said-Salim B Wickman JM Raffel SJ Kreiswirth BN Musser JM Correlation of acetate catabolism and growth yield in *Staphylococcus aureus*: implications for host-pathogen interactions Infect Immun 2003 71 4724 4732 12874354 10.1128/IAI.71.8.4724-4732.2003 PMC166023

[B49] Chatterjee I Somerville GA Heilmann C Sahl HG Maurer HH Herrmann M Very low ethanol concentrations affect the viability and growth recovery in post-stationary-phase *Staphylococcus aureus *populations Appl Environ Microbiol 2006 72 2627 2636 16597967 10.1128/AEM.72.4.2627-2636.2006 PMC1449072

[B50] Vuong C Kidder JB Jacobson ER Otto M Proctor RA Somerville GA *Staphylococcus epidermidis *polysaccharide intercellular adhesin production significantly increases during tricarboxylic acid cycle stress J Bacteriol 2005 187 2967 2973 15838022 10.1128/JB.187.9.2967-2973.2005 PMC1082835

[B51] Somerville GA Beres SB Fitzgerald JR DeLeo FR Cole RL Hoff JS Musser JM In vitro serial passage of *Staphylococcus aureus*: changes in physiology, virulence factor production, and agr nucleotide sequence J Bacteriol 2002 184 1430 1437 11844774 10.1128/JB.184.5.1430-1437.2002 PMC134861

[B52] Vossenberg JL Van de Driessen AJ da Costa MS Konings WN Homeostasis of the membrane proton permeability in *Bacillus subtilis *grown at different temperatures Biochim Biophys Acta 1999 1419 97 104 10.1016/S0005-2736(99)00063-2 10366675

[B53] Horsburgh MJ Aish JL White IJ Shaw L Lithgow JK Foster SJ Sigma(B) modulates virulence determinant expression and stress resistance: characterization of a functional *rsbU *strain derived from *Staphylococcus aureus *8325-4 J Bacteriol 2002 184 5457 5467 12218034 10.1128/JB.184.19.5457-5467.2002 PMC135357

[B54] Li D Renzoni A Estoppey T Bisognano C Francois P Kelley WL Lew DP Schrenzel J Vaudaux P Induction of fibronectin adhesins in quinolone-resistant *Staphylococcus aureus *by subinhibitory levels of ciprofloxacin or by Sigma B transcription factor activity is mediated by two separate pathways Antimicrob Agents Chemother 2005 49 916 924 15728884 10.1128/AAC.49.3.916-924.2005 PMC549254

[B55] Bisognano C Kelley WL Estoppey T Francois P Schrenzel J Li D Lew DP Hooper DC Cheung AL Vaudaux P A RecA-LexA-dependent pathway mediates ciprofloxacin-induced fibronectin binding in *Staphylococcus aureus* J Biol Chem 2004 279 9064 9071 10.1074/jbc.M309836200 14699158

[B56] Renzoni A Francois P Li D Kelley WL Lew DP Vaudaux P Schrenzel J Modulation of fibronectin adhesins and other virulence factors in a teicoplanin-resistant derivative of methicillin-resistant *Staphylococcus aureus* Antimicrob Agents Chemother 2004 48 2958 2965 15273106 10.1128/AAC.48.8.2958-2965.2004 PMC478536

[B57] Renzoni A Barras C Francois P Charbonnier Y Huggler E Garzoni C Kelley WL Majcherczyk P Schrenzel J Lew DP Vaudaux P Transcriptomic and functional analysis of an autolysis-deficient, teicoplanin-resistant derivative of methicillin-resistant *Staphylococcus aureus* Antimicrob Agents Chemother 2006 50 3048 3061 16940101 10.1128/AAC.00113-06 PMC1563528

[B58] Vaudaux P Francois P Bisognano C Li D Lew DP Schrenzel J Comparative efficacy of daptomycin and vancomycin in the therapy of experimental foreign body infection due to *Staphylococcus aureus* J Antimicrob Chemother 2003 52 89 95 10.1093/jac/dkg277 12775678

[B59] Vaudaux P Gjinovci A Bento M Li D Schrenzel J Lew DP Intensive therapy with ceftobiprole medocaril of experimental foreign-body infection by methicillin-resistant *Staphylococcus aureus* Antimicrob Agents Chemother 2005 49 3789 3793 16127054 10.1128/AAC.49.9.3789-3793.2005 PMC1195398

[B60] Clinical and Laboratory Standards Institute Methods for dilution antimicrobial susceptibility tests for bacteria that grow aerobicallyapproved standard. 7th ed. M7-A7. Wayne, PA 2006

[B61] Charbonnier Y Gettler B Francois P Bento M Renzoni A Vaudaux P Schlegel W Schrenzel J A generic approach for the design of whole-genome oligoarrays, validated for genomotyping, deletion mapping and gene expression analysis on *Staphylococcus aureus* BMC Genomics 2005 6 95 15963225 10.1186/1471-2164-6-95 PMC1183204

[B62] Scherl A Francois P Charbonnier Y Deshusses JM Koessler T Huyghe A Bento M Stahl-Zeng J Fischer A Masselot A Vaezzadeh A Galle F Renzoni A Vaudaux P Lew D Zimmermann-Ivol CG Binz PA Sanchez JC Hochstrasser DF Schrenzel J Exploring glycopeptide-resistance in *Staphylococcus aureus*: a combined proteomics and transcriptomics approach for the identification of resistance-related markers BMC Genomics 2006 7 296 17121677 10.1186/1471-2164-7-296 PMC1687195

[B63] Koessler T Francois P Charbonnier Y Huyghe A Bento M Dharan S Renzi G Lew D Harbarth S Pittet D Schrenzel J Use of oligoarrays for characterization of community-onset methicillin-resistant *Staphylococcus aureus* J Clin Microbiol 2006 44 1040 1048 16517892 10.1128/JCM.44.3.1040-1048.2006 PMC1393086

[B64] Garzoni C Francois P Huyghe A Couzinet S Tapparel C Charbonnier Y Renzoni A Lucchini S Lew DP Vaudaux P Kelley WL Schrenzel J A global view of *Staphylococcus aureus *whole genome expression upon internalization in human epithelial cells BMC Genomics 2007 8 171 17570841 10.1186/1471-2164-8-171 PMC1924023

[B65] Nagarajan V Elasri MO SAMMD: *Staphylococcus aureus *microarray meta-database BMC Genomics 2007 8 351 17910768 10.1186/1471-2164-8-351 PMC2117023

[B66] Vaudaux P Francois P Bisognano C Kelley WL Lew DP Schrenzel J Proctor RA McNamara PJ Peters G Von Eiff C Increased expression of clumping factor and fibronectin-binding proteins by *hemB *mutants of *Staphylococcus aureus *expressing small colony variant phenotypes Infect Immun 2002 70 5428 5437 12228267 10.1128/IAI.70.10.5428-5437.2002 PMC128368

