# Supplementary material for: Transcriptomic and metabolic responses of Staphylococcus aureus exposed to supra-physiological temperatures
Source: BMC Microbiol. 2009 Apr 22;9:76. doi: 10.1186/1471-2180-9-76 (PMC2687450; doi:10.1186/1471-2180-9-76)
Supplement: Additional file 5 — Sequences of primers and TaqMan probes used in this study. [file 1471-2180-9-76-S5.doc]

**Sequences of primers and TaqMan probes used in this study**

| **Primer or probe^a^** | **Oligonucleotide sequence** |
| --- | --- |
| ctsR-14F | 5’-CTGACATCATAGAACAATACATCAAACG-3’ |
| ctsR-110R | 5’-CAATCAAAACGCTGTGCGATA-3’ |
| ctsR-(86-56)-P | 5’-GCTCTCTGAATTTCAACGACATCTTCATTCG-3’ |
|  |  |
| mcsA-309F | 5’-TGG GTG TGC TAA TTG TTA TGC AA-3’ |
| mcsA-398R | 5’-TTT CCA ACG TGC TCA AAT TGT C-3’ |
| mcsA-344-P | 5’-ACA TCA TTG ATA TCG TCC GCA GAG TTC AAG GT-3’ |
|  |  |
| mcsB-568F | 5’-AAT CGT TTT GGA TAT ACA ATC AGA GGT A-3’ |
| mcsA-659R | 5’-CCA AGT GTA AGT TGG TTG GAT ACT TG-3’ |
| mcsB-P | 5’-TGT CCA TAA ACT TGC GAA CCT TCA CCG-3’ |
|  |  |
| clpC-311F | 5’-GAA CGG AAC ATA TTT TAT TAG GCT TGA-3’ |
| clpC-443R | 5’-CTC ATT TCA GGG TTT CCT AAA GCT T-3’ |
| clpC-P | 5’-TCT TGC TGC AAC ACC TTC ATT TTC ACG A-3' |
|  |  |
| clpP-316F | 5’-GCT GGT GCA AAA GGT AAA CGT T-3’ |
| clpP-392R | 5’-CCT TGA GCA CCA CCT AAT GGT T-3’ |
| clpP-339-P | 5’-CGC GTT ACC AAA TGC AGA AGT AAT GAT TCA C-3’ |
|  |  |
| hrcA-39F | 5’-TGT TGA GGA TTA TGT TGA TTT TGG A-3’ |
| hrcA-132R | 5’-ATT TCT AAT TGT AGC AGG ACT AAC ATT CA-3’ |
| hrcA-66-P | 5’-ACC CGT TGG TTC TAA AAC ACT AAT TGA GCG ACA T-3’ |
|  |  |
| dnaK-1009F | 5’-CCG GAC GAA GTA GTG GCA AT-3’ |
| dnaK-1136R | 5’-TTC ATA CGT CCA CCT AAA ATT TCA AT-3’ |
| dnak-P | 5’-TGA TAA CGC CAC CTT GGA TTG CAG CT-3’ |
|  |  |
| groEL-1350F | 5’-AAT TGC TGA AAA TGC AGG ATT AGA-3’ |
| groEL-1437R | 5’-TGT AGC AGC GTT AAA ACC AAC AC-3’ |
| groEL-1384-P | 5’-ATT GTA GAA CGT TTG AAA AAC GCA GAG CCG-3’ |

^a^Forward (F) and reverse (R) primers and the TaqMan probe (P) for the ORFs are shown.
