# Supplementary material for: Transcriptomic and metabolic responses of Staphylococcus aureus exposed to supra-physiological temperatures
Source: BMC Microbiol. 2009 Apr 22;9:76. doi: 10.1186/1471-2180-9-76 (PMC2687450; doi:10.1186/1471-2180-9-76)
Supplement: Supplementary file 4 [file 1471-2180-9-76-S1.doc]

Table 1: COG functional categories of genes whose transcript levels showed ≥2-fold changes after 10 min heat shock

	48°C		43°C	
	up	down	up	down
Translation	3	15	1	8
Transcription	10	4	6	0
Replication, recombination/repair	9	0	0	0
Cell cycle control	4	1	2	0
Defense mechanisms	3	1	0	1
Signal transduction mechanisms	1	4	0	0
Cell wall/membrane biogenesis	3	10	0	3
Posttranslational modification, chaperones	8	5	8	1
Energy production/conversion	5	14	0	7
Carbohydrate transport/metabolism	20	6	2	0
Amino acid transport/metabolism	31	24	4	7
Nucleotide transport/metabolism	1	26	0	1
Coenzyme transport/metabolism	8	6	0	1
Lipid transport/metabolism	0	8	0	2
Inorganic ion transport/metabolism	13	14	4	3
General function prediction only	63	18	9	2
Function unknown	118	76	18	3
Total	300	232	54	39
