# Supplementary material for: Transcriptomic and metabolic responses of Staphylococcus aureus exposed to supra-physiological temperatures
Source: BMC Microbiol. 2009 Apr 22;9:76. doi: 10.1186/1471-2180-9-76 (PMC2687450; doi:10.1186/1471-2180-9-76)
Supplement: Supplementary file 5 [file 1471-2180-9-76-S3.doc]

Table 2: Evaluation by microarray and qRT-PCR of the transcriptional responses of *S. aureus* heat stress regulons[a]

			43°C							48°C						
			ISP794					ISPU		ISP794					ISPU[b]	
			Microarray			qRT-PCR				Microarray			qRT-PCR			
SA Number	Name	Description	Probe intensity	Mean of ratio	SEM	Fold change	SEM	Fold change	SEM	Probe Intensity	Mean of ratio	SEM	Fold change	SEM	Fold change	SEM
SA0480	*ctsR*	class III stress gene repressor	◆◆◆◆	5.41	0.23	7.73	0.91	7.36	2.63	◆◆◆◆	4.93[c]	0.94	16.34	1.29	22.94	6.91
SA0481	*mcsA*	modulator of CtsR repression	◆◆◆◆	4.91	0.45	5.9	0.17	6.41	0.41	◆◆◆◆	5.71[c]	1.09	12.13	0.69	15.24	0.98
SA0482	*mcsB*	modulator of CtsR repression	◆◆◆◆	5.74	0.04	5.58	0.57	5.39	0.59	◆◆◆◆	7.75[c]	1.61	13	2.14	13.45	2.11
SA0483	*clpC*	Clp ATPase	◆◆◆◆	5.09	0.27	3.86	0.61	7.26	1.68	◆◆	11.21	0.84	9.51	1.12	16.11	4.71
SA0835	*clpB*	Clp ATPase	◆◆◆◆	5.60	1.11	ND[d]	ND	ND	ND	◆◆◆◆	6.99[c]	1.53	ND	ND	ND	ND
SA0723	*clpP*	ATP-dependent Clp protease	◆◆◆◆	2.99	0.45	4.08	0.39	5.06	0.72	◆◆◆	3.17	0.17	6.36	0.6	8	0.69
SA1411	*hrcA*	transcriptional repressor	◆◆◆◆	4.98	0.11	3.94	0.25	4.29	0.47	◆◆◆◆	4.31[c]	0.18	8.51	1.47	8.17	0.47
SA1410	*grpE*	GrpE protein	◆◆◆◆	2.85	0.18	ND	ND	ND	ND	◆◆◆◆	2.06[c]	0.53	ND	ND	ND	ND
SA1409	*dnaK*	DnaK protein	◆◆◆◆	3.85	0.04	2.99	0.35	5.54	0.36	◆◆◆◆	6.27[c]	1.16	7.06	0.72	8.28	0.78
SA1408	*dnaJ*	DnaJ protein	◆◆◆◆	4.49	0.26	ND	ND	ND	ND	◆◆◆◆	3.33[c]	1.02	ND	ND	ND	ND
SA1407	*prmA*	ribosomal protein L11 methyltransferase	◆◆◆	2.81	0.26	ND	ND	ND	ND	◆◆◆	6.27	0.18	ND	ND	ND	ND
SA1836	*groEL*	GroEL protein	◆◆◆◆	3.87	0.31	3.1	0.88	6.5	1.18	◆◆◆◆	6.08	0.06	5.35	0.35	9.58	1.27
SA1837	*groES*	GroES protein	◆◆◆◆	6.42	0.41	ND	ND	ND	ND	◆◆◆◆	4.87[c]	0.07	ND	ND	ND	ND
SA0879	*htrA*	serine protease HtrA	◆◆	0.87	0.01	ND	ND	ND	ND	◆◆	0.33	0.01	ND	ND	ND	ND

[a]*S. aureus* ISP794 or ISPU exposed for 10 min heat shock at 43°C or 48°C

[b]$RsbU^+$-restored derivative of ISP794

[c]Saturated signal on the microarray scanner

[d]ND: not determined
